# A Virtual Reality–Based Cognitive Defusion Application for Youth Depression and Anxiety: Mixed Methods Experimental Study

**DOI:** 10.2196/70160

**Published:** 2025-08-07

**Authors:** Imogen H Bell, Cassandra Li, Andrew Thompson, Carli Ellinghaus, Shaunagh O’Sullivan, Kate Alexandra Reynolds, Greg Wadley, Yang Liu, Sarah Bendall, John Gleeson, Lucia Valmaggia, Mario Alvarez-Jimenez

**Affiliations:** 1 Orygen Parkville Australia; 2 Centre for Youth Mental Health The University of Melbourne Parkville Australia; 3 Division of Mental Health and Wellbeing University of Warwick Warwick United Kingdom; 4 School of Computing and Information Systems The University of Melbourne Melbourne Australia; 5 Healthy Brain and Mind Research Centre and School of Behavioural and Health Sciences Australian Catholic University Melbourne Australia; 6 Institute of Psychiatry, Psychology and Neuroscience King's College London London United Kingdom

**Keywords:** virtual reality, depression, anxiety, third-wave psychological treatment, mindfulness, cognitive defusion, youth mental health

## Abstract

**Background:**

Third-wave psychological treatments such as acceptance and commitment therapy can be effective for improving depression and anxiety in youth. However, third-wave therapeutic techniques such as cognitive defusion can be abstract, challenging to learn, and difficult to apply in real-world settings. Translating these techniques into virtual reality (VR) may provide interactive, enjoyable, and concrete learning opportunities, potentially enhancing engagement and effectiveness. This study evaluated a novel VR application that translates the technique of cognitive defusion into a brief, gamified VR experience.

**Objective:**

The objectives of this study were to evaluate the feasibility, acceptability, usability, and safety of the VR cognitive defusion application; examine whether it could improve negative thinking and mood states; and understand how it compared to a non-VR cognitive defusion exercise.

**Methods:**

In a mixed methods experimental study, 20 young people completed both a VR and audio cognitive defusion exercise in a randomized order within a single session. Quantitative state-based measures were taken before and after each exercise, and a qualitative interview at the end focused on how the two experiences compared.

**Results:**

It was feasible to recruit participants, and all participants completed both exercises and assessments. Both the VR and audio exercises were acceptable to participants, with qualitative themes highlighting a preference for VR due to the novel and engaging format; however, there was a need for better guidance and more personalized environments. No severe adverse events were reported, although one participant experienced distress during the VR exercise. Pretest-posttest effects showed improvements in thought discomfort, cognitive defusion, and state anger for both the VR and audio conditions (*P*<.05), with the latter showing broader improvements, including thought negativity, rumination, tension, depression, distress, and confusion (*P*<.05).

**Conclusions:**

The VR cognitive defusion application was feasible, safe, and acceptable for young people, with potential to enhance mental health treatment through an engaging and enjoyable approach to learning third-wave cognitive behavioral therapy techniques. While VR was preferred by participants, further refinements could improve effectiveness. Future research should focus on enhancing the VR application design based on user feedback, incorporating audio guidance, and conducting a larger trial in real-world settings to thoroughly evaluate the effectiveness and implementation of the VR application.

## Introduction

### Background

Virtual reality (VR) is an emerging technology with the potential to transform mental health treatment [1-3]. VR immerses users in a virtual environment through a headset, allowing for real-time interaction with virtual objects via hand controllers or motion sensors. The ability to tailor and control experiences within VR offers a unique opportunity to enhance mental health interventions by delivering treatments in personalized, simulated real-world environments that provide strong ecological validity [2]. While meta-analyses have supported the efficacy of VR treatments for various mental disorders in adults [2,4], its application in youth mental health remains critically underexplored [5,6].

Mental disorders emerge before the age of 25 years in 75% of cases [7,8], representing a critical time for early intervention [9]. Anxiety and depression are the most prevalent disorders among youth, with 1 in 4 young people meeting the diagnostic criteria [10]. Despite high rates of mental ill-health among young people, current treatments remain limited, with small treatment effect sizes, poor engagement, and high relapse rates [11-13]. VR offers innovative solutions to these challenges by providing a potentially engaging, effective, and accessible vehicle for treatment [1]. Young people widely use digital technologies in their day-to-day lives and have shown interest in VR for mental health support [14]. With consumer adoption of VR technology predicted to rise over the coming years [15], it is timely to expand the existing evidence base and explore the therapeutic potential of this technology for young people with mental ill-health.

The past decade has seen a rapid increase in the number of studies exploring the use of VR for the treatment of mental disorders [2,16]. Most of this research has focused on VR-based cognitive behavioral treatments (VR-CBTs) [16]. VR-CBT typically involves exposure to anxiety-inducing stimuli in virtual environments guided by a clinician (eg, Pot-Kolder et al [17]). Randomized controlled trials have provided robust evidence supporting the efficacy of VR-CBT in improving outcomes in anxiety disorders, psychosis, eating disorders, and trauma-related conditions in adults (see Bell et al [2] for a review). However, relatively limited research has explored the potential therapeutic applications of VR beyond exposure-based cognitive behavioral therapy (CBT) approaches, and research in youth is highly limited [2,5,6,16].

Third-wave psychological treatments such as acceptance and commitment therapy (ACT), dialectical behavior therapy, and mindfulness-based cognitive therapy [18] may be particularly well suited to VR. While second-wave CBT focuses on modifying maladaptive thoughts that drive problematic behaviors, third-wave approaches focus on adapting the relationship between the individual and their mental processes as well as targeting broader values and life goals [18]. Third-wave approaches typically use experiential techniques to shift the dynamic between the individual and their mental experiences to foster more adaptive and flexible ways of responding to them. For example, cognitive defusion, a core technique within ACT [19-21], aims to help individuals separate themselves or “defuse” from distressing thoughts and emotions, often using experiential and conceptual techniques such as metaphors and visualization. In the context of psychological treatment, cognitive defusion exercises typically involve a therapist guiding individuals to imagine negative thoughts in ways that are incongruent with their existing emotional relations with them (eg, singing the negative thought to the tune of “Happy Birthday”). By viewing and relating to these thoughts in nonliteral and often paradoxical ways, individuals can shift the negative associations they have with these thoughts and “defuse” or “decenter” from them, viewing them as fleeting and nonthreatening rather than literal facts. The process of decentering has been highlighted as a core “active ingredient” underpinning effective interventions for youth depression and anxiety [22,23], particularly in fostering objective and flexible ways of responding to negative thoughts and emotions.

Third-wave psychological treatments have demonstrated effectiveness in improving depression and anxiety in youth and adults [24-26]. However, while core techniques such as cognitive defusion can be highly effective for some individuals [19,21,27], young people can find it challenging to conceptualize their internal experiences in abstract ways, limiting the effectiveness of the technique and engagement with treatment [28]. Recognizing this, Halliburton and Cooper [28] emphasize the importance of adapting ACT for the developmental needs of adolescent populations. To accommodate emerging cognitive development in adolescence, the authors recommend that techniques be brief, simple and concrete, highly interactive, and experiential. The use of creative tools and activities can also promote engagement. VR technology is highly suited to supporting these adaptations by transforming the way in which these techniques are taught and learned. Immersive environments enable individuals to directly observe and interact with their mental processes through virtual representations, bypassing the need for abstract thinking and visualization. This approach facilitates skill mastery through playful, low-risk encounters and may boost the positive effects of techniques such as cognitive defusion by translating them from abstract and conceptual into something more literal and concrete. Furthermore, by learning and practicing these skills within personally relevant virtual environments, young people may be more likely to apply these skills in everyday life. Harnessing gamification in VR environments to create a more motivating, fun, and low-risk learning experience could also enhance the goal of these techniques to change the nature of the relationship between the individual and their thoughts while also addressing barriers to treatment engagement. When used as a blended tool [29], clinicians could use VR within sessions to guide young people to learn and practice third-wave skills in safe and controlled VR environments to facilitate their translation into real-world settings. In addition, young people could use the intervention independently to manage and cope with mental health challenges as they arise. Such brief, flexible, and engaging VR-based therapeutic tools have the potential to enhance engagement and treatment efficacy as well as increase access to on-demand support in daily life.

A study by Prudenzi et al [30] investigated a VR-based cognitive defusion task in a nonclinical sample of young adults and found that the task reduced the believability and discomfort associated with negative thoughts. A small number of studies have also examined the use of VR for related techniques such as mindfulness [31-34], with preliminary evidence of positive outcomes. However, this research is at the very early stages with predominantly single case studies and typically involves populations of adults without a mental health condition. Furthermore, while many claim that VR has advantages over and above standard face-to-face therapeutic approaches, limited research has been conducted to test this empirically. This research is critically needed to provide a strong foundation for ongoing development of VR-based interventions for youth mental health.

### Study Aims

To investigate the potential of VR for supporting third-wave treatment of youth depression and anxiety, a prototype application was developed that translated the technique of cognitive defusion (the primary target mechanism) into a personalized, immersive, gamified VR experience. The aims of this mixed methods experimental study were to (1) evaluate the feasibility, acceptability, usability, and safety of the VR cognitive defusion application; (2) explore whether the VR cognitive defusion application could reduce negative thinking and improve mood states; and (3) gather feedback from young people on how the VR cognitive defusion application experience compared to a non-VR audio-guided cognitive defusion exercise.

## Methods

### Study Design

The study design was a crossover, repeated measures, mixed methods experimental study. This involved all participants being exposed to 2 forms of cognitive defusion exercises sequentially and in a randomized order: one through a VR application and the other via a traditional audio exercise. Exposing all participants to both exercises in a randomized order enabled the examination of how the two compared.

### Participants

Participants (N=20) included young people who were recruited via *headspace* services across Metropolitan Melbourne, Victoria, Australia. *headspace* is a nationwide organization delivering mental health treatment for youth aged 12 to 25 years across Australia. Current and previous clients of 4 *headspace* centers in the metropolitan region of northwest Melbourne were approached for the study. The sample size was deemed appropriate to meet the primary aim of assessing the acceptability, usability, and safety of the VR intervention, as well as exploring whether there were preliminary effects on state-based outcomes [35]. Informed consent was obtained from participants before their commencement, with additional parent or guardian consent obtained for participants aged <18 years. Participants were eligible to take part if the following inclusion criteria were met: (1) aged between 16 and 25 years, (2) normal or corrected-to-normal vision and hearing, (3) clinical levels of depression or anxiety (score of ≥10 on the 8-item Patient Health Questionnaire [PHQ-8] [36] or 7-item Generalized Anxiety Disorder scale [GAD-7] [37]), (4) high levels of repetitive negative thinking (RNT; score of ≥15 on the Brief Penn State Worry Questionnaire (Brief PSWQ) or ≥9 on the Brief Ruminative Response Scale (Brief RRS) [38]), and (5) sufficient command of the English language.

Exclusion criteria were (1) inability to provide informed consent, (2) history of photosensitive epilepsy or previous experience of severe simulator sickness, and (3) being a psychiatric inpatient or under the care of a mental health crisis team.

### Procedure

#### Overview

Participants were screened for eligibility over the phone, with those eligible then booked for the in-person consent and testing session. This 90-minute session involved informed consent and a baseline assessment followed by the experimental procedure.

The experimental procedure involved being allocated to complete one of two possible intervention sequences via simple randomization: (1) VR defusion exercise followed by audio defusion exercise or (2) audio defusion exercise followed by VR defusion exercise. Both exercises were matched in terms of duration and took approximately 10 minutes each to complete. The session was conducted in a private clinical space with a researcher.

#### Pretest-Posttest Assessments

Participants completed measures at baseline (T1); after they completed either the VR or audio exercise first (T2); and again following their completion of the alternate exercise, VR or audio (T3). Following T3 questionnaires, participants completed a brief qualitative feedback interview of their experience with a research assistant, which lasted an average of 8 (range 4-26) minutes.

#### Explanation of Cognitive Defusion

Regardless of which order the exercises were completed in, participants were provided with a short explanation of cognitive defusion and how it can help people disconnect from their distressing thoughts before completing the first exercise (adapted from an ACT manual [39,40]). This was delivered by the researcher using a script (Multimedia Appendix 1).

#### Rumination Induction

Following the protocol by Masuda et al [21], before each exercise, a brief rumination induction procedure took place in which participants were prompted to choose and reflect on a negative thought that had been distressing them recently and keep this in mind during the exercises. This procedure was embedded within the Negative Thought Assessment (NTA) [30,41,42] described in the Measures section.

### Conditions

#### Overview

In total, 2 experimental conditions were designed for this study: the VR exercise and an audio exercise.

Both experimental conditions were designed to guide users through 2 different forms of cognitive defusion, as described in the Introduction section. The 2 conditions were informed by practices commonly used in ACT, the approach from which defusion was developed [39,40].

#### VR Condition

The VR application was preloaded onto an Oculus Quest 2 VR headset for the purpose of this study. The research assistant conducting the session invited participants to wear the VR headset and explained how to use the hand controllers. Participants were informed at the start that they could stop the exercise at any time if they became distressed or uncomfortable. They were encouraged to complete the exercise from a standing position to allow for range of movement, but an option for completing the exercise in a seated position was also available. Before beginning the exercise, a verbal check of visual and audio clarity was conducted by the researcher.

**Figure 1:**
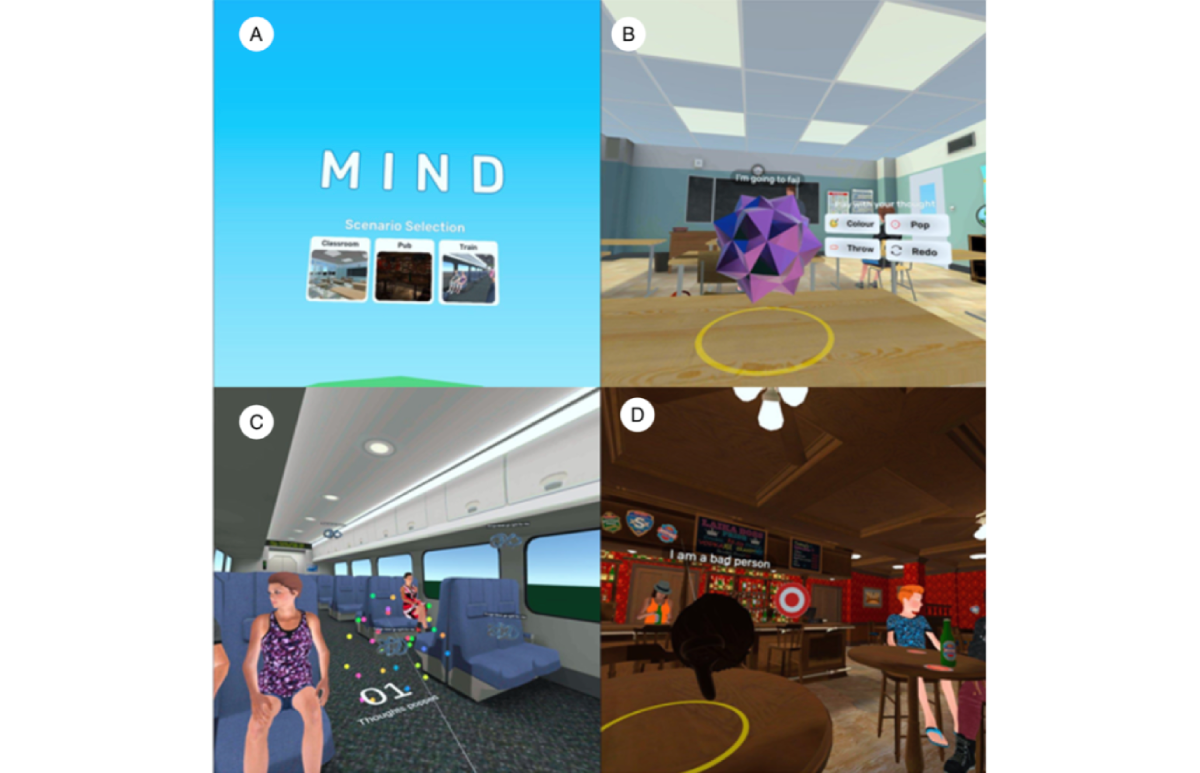
Scenes from the virtual reality cognitive defusion application.

**Figure 2:**
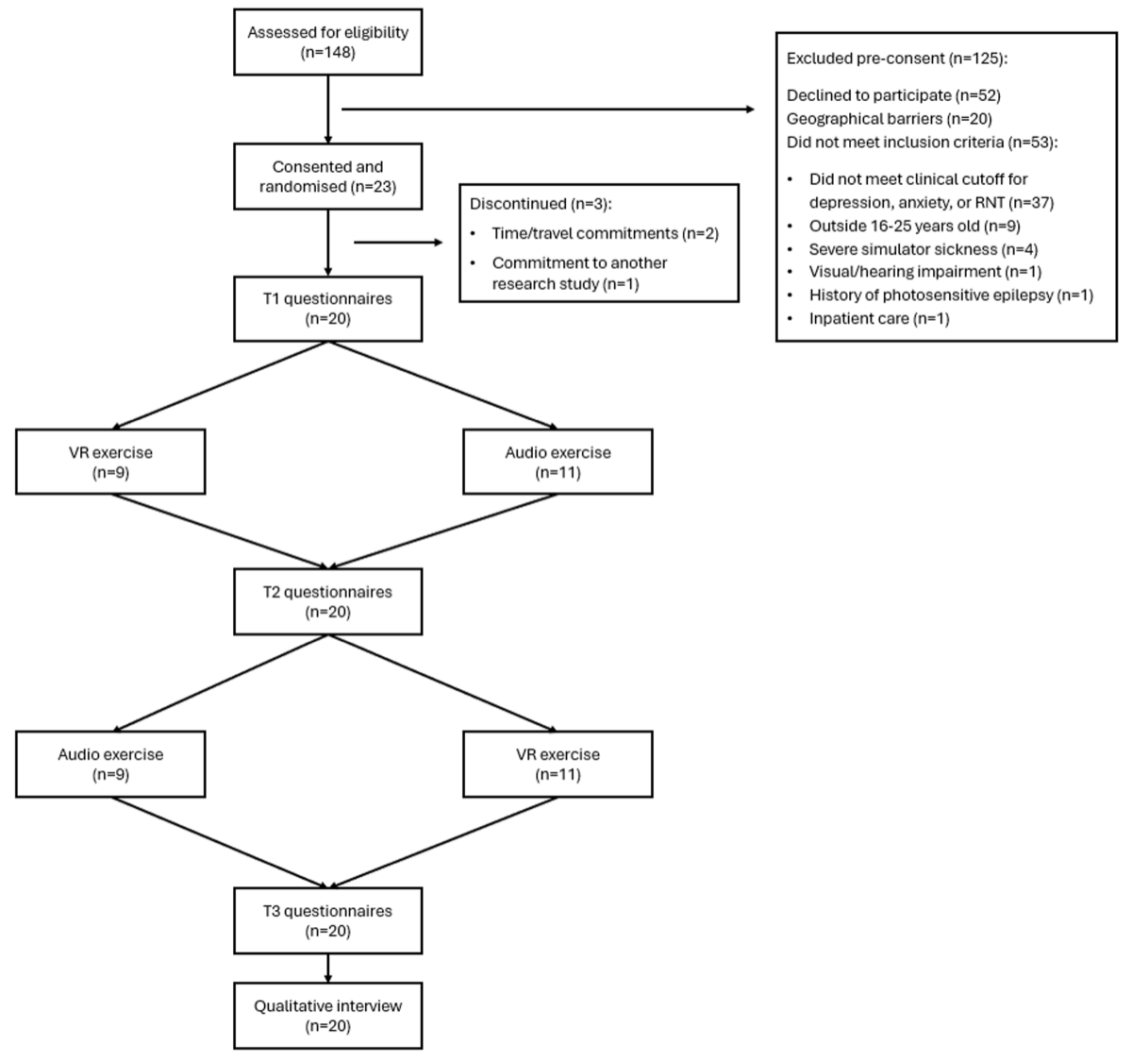
Study flow diagram illustrating participant recruitment to completion. RNT: repetitive negative thinking; VR: virtual reality.

When participants launched the VR application, they were presented with a menu screen showing 3 different environments to choose from: a classroom, a pub, and a train (Figure 1A). These environments were designed based on user consultations with young people with lived experience of mental health difficulties and clinicians that highlighted these as common environments where negative thoughts were experienced. Participants were encouraged to choose the environment in which they felt that their negative thoughts were most likely to occur. When each participant entered the environment, they were faced with an animated object and instructed to imagine this as representing the thought they had been struggling with. They were then guided to customize the thought by typing it in using a virtual keyboard presented to them within the scene or selecting from prefilled thought prompts (eg, “I’m going to fail,” “I am being judged,” or “I am a bad person”). The thought was then represented in the environment as text above the animated object (Figure 1B). Participants were then instructed to interact with their chosen thought and manipulate it in different ways using the hand controllers. These options included customizing its shape and color, picking it up and moving it around in the environment, and making the thought float upward above them. Among the options were also two games that they could play with the thought object: (1) “popping” the thought objects as they appeared around them to receive points (Figure 1C) or (2) throwing the thought at targets that appeared in the environment to receive points (Figure 1D).

Throughout the exercise, participants were guided by the researcher. The researcher followed a verbal script (Multimedia Appendix 1) supporting the participant in exploring their negative thought in a visual form with curiosity and acceptance. The verbal script mirrored the audio defusion script with slight variations to accommodate the VR experience. The full experience lasted 10 minutes. Once the exercise was finished, participants were prompted to remove the VR headset.

#### Audio Condition

The audio condition was designed to use the same defusion principles and identical instructions but in a non-VR format using the participant’s imagination. In a traditional psychological treatment setting, clinicians would typically guide the individual through the exercise following a verbal script spoken aloud in the session. For the purposes of this study, a standard prerecorded guided audio exercise was used (see Multimedia Appendix 1 for the script). In the exercise, the participant was guided by a single voice without any background noise or effects. The voice instructed the listener to identify a target negative thought and think about it in different ways, such as imagining their thought as having different colors, shapes, forms, and contexts. These interactions were designed to match those of the VR exercise in that individuals were guided to think of environments where they were likely to experience negative thoughts and then instructed to visualize interacting with them in the same ways using their imagination. Participants listened to the audio exercise via headphones through an iPad device, remaining in a seated position, with the researcher remaining in the room to monitor for any signs of distress. The length of the audio exercise was 10 minutes to match the VR exercise.

### Measures

The protocol for this study was developed in collaboration with young people with lived experience of a mental health condition. Questionnaire completion at each time point took approximately 10 minutes, and no participants reported discomfort, fatigue, or disengagement.

#### Demographics and Technology Use

Basic demographic information was collected, including age, gender, country of birth, Aboriginal or Torres Strait Islander origin, languages spoken at home, current living situation, and which *headspace* service they were a client of.

Questionnaires were used to collect experiences with technology use, including whether they owned a smartphone, laptop, or desktop computer and how many hours per day they used each owned device. Participants were asked about the ways in which they had sought or engaged with health support in the past (eg, email, phone, or online information). Previous experience with VR use was also collected. Those who reported previous use were prompted to complete additional items addressing the frequency of use (9-point scale ranging from “Very rarely*”* to “Several times an hour”) and how comfortable they felt using VR (5-point scale ranging from “Very uncomfortable” to “Very comfortable”), as well as indicating whether they had used VR for therapeutic reasons (eg, mindfulness, relaxation, and exposure).

These measures were completed at baseline (T1).

#### Feasibility, Acceptability, Usability, and Safety

##### Feasibility

Feasibility was measured via recruitment and retention rates, as well as completion of study assessments and procedures.

##### Acceptability and Usability

A 14-item user experience questionnaire designed specifically for this study was used to evaluate and compare the usability and acceptability of both the VR and audio conditions. The questionnaire was designed to assess different aspects of the participants’ experiences with the exercises. Respondents answered statements (eg, “I found the experience enjoyable”) by rating their experience on a 5-point Likert scale ranging from 1 (“strongly disagree”) to 5 (“strongly agree”), with higher scores indicating better user experience and acceptability. This measure was completed after each defusion exercise (T2 and T3).

##### ITC–Sense of Presence Inventory

This is a 44-item scale measuring sense of presence within a virtual environment. This measure captures the degree to which the user feels physically present in the virtual environment, the level of engagement, and negative effects (eg, motion sickness and eye strain). Respondents answer statements (eg, “I felt sad that my experience was over”) using a 5-point Likert scale ranging from 1 (“strongly disagree”) to 5 (“strongly agree”), with higher scores indicating greater user experience and acceptability. The ITC–Sense of Presence Inventory (ITC-SOPI) has been validated in adult populations and is commonly used in VR research [43]. This measure was completed following the VR condition only (T2 or T3 depending on the exercise order).

##### Safety

Safety was measured by monitoring potential adverse events during both exercises, responses to the negative effects, questions in the user experience questionnaire and ITC-SOPI [43], and questions in a qualitative interview described later in this section.

#### Clinical Measures

Participant characteristics were assessed using the following measures, which were completed at baseline (T1).

#### PHQ-8 Measure

The PHQ-8 is an 8-item scale commonly used to screen for depressive disorders. Respondents indicate the frequency of depressive symptoms experienced over the previous 2 weeks on a 4-point Likert scale ranging from 0 (“not at all”) to 3 (“nearly every day”), where higher scores indicate greater severity of depression. Scores are summed, with scores of ≥10 indicating moderate to severe depressive symptoms. The PHQ-8 [36] has demonstrated good reliability and validity with adolescent populations [44].

#### GAD-7 Measure

The GAD-7 is a 7-item scale commonly used as a screener for anxiety disorders. Respondents indicate the frequency of symptoms of anxiety over the previous 2 weeks on a 4-point Likert scale from 0 (“not at all”) to 3 (“nearly every day”), where higher scores indicate greater severity of anxiety. Scores are summed, with scores of ≥10 indicating moderate to severe levels of anxiety. The GAD-7 has good reliability and construct validity with adolescent populations [37].

#### Brief PSWQ Measure

The Brief PSWQ is a 5-item scale measuring tendencies toward worry. Respondents rate statements (eg, “Many situations make me worry”) on a 5-point Likert scale from 1 (“not typical of me”) to 5 (“very typical of me”), with higher scores indicative of greater trait worry. The scale has been validated in an adult population with good reliability and validity [38].

#### Brief RRS Measure

The Brief RRS is a 5-item scale measuring tendencies toward rumination. Respondents are asked to indicate how often they ruminate when they feel down, sad, or depressed. They indicate their response using a 4-point Likert scale ranging from 1 (“almost never”) to 4 (“almost always”), with higher scores equating to higher degrees of ruminative symptoms. This measure has shown good reliability and validity [38].

#### Repetitive Thinking Questionnaire–10

The Repetitive Thinking Questionnaire–10 (RTQ-10) is a 10-item scale used to measure tendencies toward RNT (a trait capturing the process of rumination and worry). Respondents rate statements capturing their tendency to engage in repetitive thinking when they feel distressed or upset using a 5-point Likert scale ranging from 1 (“not at all”) to 5 (“very true”), with higher scores reflecting greater tendencies toward RNT. The RTQ-10 has demonstrated robust internal consistency and convergent and divergent validity [45].

#### Therapeutic Process Measures

##### NTA Measure

The NTA scale was completed as part of the rumination induction procedure to elicit rumination and capture state-based responses to this experience. The NTA has been used in previous experimental studies to elicit rumination and measure the immediate effects of cognitive defusion exercises [30,41,42]. This measure prompts respondents to generate and reflect on a particular negative thought that they identify as upsetting, find hard to stop thinking about, or bothers them a lot. Respondents then rate the degree of discomfort (ie, “How uncomfortable is the thought”) and believability (ie, “How accurate or true do you think the thought is?”) and the perceived importance of the thought (“How important is it to you not to have this thought?”). One item used in the study by Prudenzi et al [30] was added to measure the degree of negativity of the thought (“How negative is the thought?”). All items are rated on a 10-point visual analogue scale ranging from 0 (not at all uncomfortable/accurate or true/important/negative) to 10 (very uncomfortable/accurate or true/important/negative). This measure was completed at baseline (T1) and following the first exercise (T2).

##### Toronto Mindfulness Scale–State Version

Toronto Mindfulness Scale (TMS)–State Version [46] is a 13-item scale measuring state mindfulness. Items are presented as statements referring to 2 constructs, decentering and curiosity, which are each scored as subscales. Decentering refers to psychological distance from thoughts, and curiosity refers to appreciation of thoughts with an accepting and curious attitude. Responses are scored on a 5-point Likert scale ranging from 0 (“not at all”) to 4 (“very much”), with higher scores indicating greater state mindfulness. The scale has demonstrated good reliability and validity, with good sensitivity to change over time [47]. This measure was completed following the first exercise (T2).

##### State Cognitive Fusion Questionnaire

The State Cognitive Fusion Questionnaire (SCDQ) is a 7-item scale measuring states of cognitive defusion. Items include statements referring to the experience of struggling or being entangled with distressing thoughts in the present moment. Respondents indicate their response on a 7-point Likert scale from 1 (“not at all true”) to 7 (“completely true”), with higher scores indicating greater levels of defusion. The SCDQ has demonstrated good internal consistency and construct validity in student samples [48]. This measure was completed at baseline (T1) and following the first exercise (T2).

##### Brief State Rumination Inventory

The Brief State Rumination Inventory (BSRI) [49] is an 8-item scale measuring state depressive rumination. Respondents indicate the extent to which each statement describes their feelings and thoughts in the immediate moment (eg, “Right now, I wonder why I react the way I do”) on a 100-point visual analogue scale ranging from 0 (“completely disagree”) to 100 (“completely agree”), where higher scores indicate greater state rumination. The scale has demonstrated good reliability and validity. This measure was completed at baseline (T1) and following the first exercise (T2).

#### Mood State Measures

##### Profile of Mood States–Adolescents

The Profile of Mood States–Adolescents (POMS-A) is a 24-item scale where respondents indicate how they feel in the moment across different mood items (eg, sleepy, energetic, and confused) using a 5-point Likert scale ranging from 1 (“not at all”) to 5 (“extremely”). Higher scores indicate greater overall mood disturbance. This scale has demonstrated good reliability and criterion validity in the assessment of mood in adolescent populations [50,51]. This measure was completed at baseline (T1) and following the first exercise (T2).

##### Subjective Units of Distress Scale

The Subjective Units of Distress Scale (SUDS) [52] is a 1-item scale in which respondents are asked to rate the level of anxiety that they are currently experiencing using a slider scale, with scores ranging from 0 (“no anxiety, calm”) to 100 (“very severe anxiety, worst ever experienced”). The scale is commonly used to measure states of distress during therapeutic exercises, with a higher score indicating greater state distress. This measure was completed at baseline (T1) and following the first exercise (T2).

#### Qualitative Interview

A brief semistructured qualitative interview was conducted by the research assistant at the end of the experimental study that inquired about the following:

- What was your experience of the VR exercise?
- How did the VR experience compare to the audio exercise?
- Which did you prefer out of the VR experience and audio exercise and why?
- Imagine that the VR exercise was taught to you in therapy by a psychologist:
  Do you think there would be any benefits to the VR exercise over the audio exercise? Can you explain your answer?
  Do you think there would be any downsides to the VR exercise over the audio exercise? Can you explain your answer?
- Would you recommend the VR experience to other young people with difficult thoughts and why or why not?
- Is there anything you would change about the VR experience to make it better?

### Analysis

#### Quantitative Data

Descriptive statistics were used to report the feasibility, acceptability, usability, and safety of the VR and audio conditions. Independent 2-tailed *t* tests and chi-square tests were conducted to test for differences between groups in baseline demographic and clinical characteristics. Repeated measures *t* tests and within-group effect sizes (Cohen *d*) were reported for changes in pre- to postexercise scores on state-based measures for the VR and audio groups. Only T1 and T2 scores on state-based measures within groups were compared (ie, pretest-posttest changes before and after the VR or audio exercises depending on the order) to avoid carryover effects into the second exercise. The primary measure of interest was the within-group effect size and associated CIs. The sample size was not powered to detect statistically significant differences between conditions but, instead, was selected to provide sufficient data to evaluate feasibility and inform understanding of whether the approach could engage target mechanisms and influence mood states as a proof of concept. This is a critical step in intervention development and validation, particularly in emerging areas such as VR-based therapy, and our study design reflects recommendations for pilot trials with small sample sizes [35].

#### Qualitative Data

Qualitative data collected from interviews with participants were analyzed using reflexive thematic analysis guided by the approach by Braun and Clarke [53]. This method was chosen for its flexibility and suitability in exploring participants’ subjective experiences. In total, 2 researchers (IHB and CL) independently read and coded an initial subset of 20% (4/20) of the transcripts to familiarize themselves with the data and inductively generate initial codes. They then met to discuss their interpretations, reflexively exploring their assumptions and positionalities, and developed a shared, flexible coding framework. The remaining transcripts (16/20, 80%) were coded using this framework, with regular discussions to review coding decisions.

Themes were developed through an iterative process of data engagement, coding, and theme refinement following the principles of reflexive thematic analysis. The final themes represent patterns of shared meaning relevant to the research question and were refined for coherence, internal consistency, and analytic depth. To enhance credibility and dependability, researcher triangulation, regular peer debriefing, and an audit trail of coding and theme development decisions were used. Reflexivity was maintained throughout via memos and ongoing discussion of interpretive lenses. Confirmability was supported by maintaining transparency in our analytic process and grounding the themes in rich data extracts. While we did not aim for statistical generalizability, sufficient contextual detail was extracted to support transferability to similar settings.

### Ethical Considerations

This project was approved by the University of Melbourne Human Research Ethics Committee (ID 2056403.1). Participants provided written informed consent. All participants received a full explanation of the study in lay terms related to the aims, study procedures, and potential risks and benefits in taking part before providing informed consent. The participants were reminded that they could withdraw from the study at any time without prejudice. Participants were provided with a copy of the participant information and consent form and were reimbursed Aus $45 (US $28.94) for taking part in the study. All data were stored in a deidentified format within password-protected electronic files only accessible to the research team.

## Results

### Feasibility and Sample Characteristics

Figure 2 shows a CONSORT (Consolidated Standards of Reporting Trials) diagram of the recruitment and testing process. Of the 148 potential participants who were assessed for eligibility for the study, 95 (64.2%) were eligible. Of the 95 eligible participants, 23 (24%) were interested and completed consent procedures. In total, 13% (3/23) of the participants dropped out between consenting and the baseline assessment, with a total of 20 participants being randomized (n=9, 45% to the VR-first condition and n=11, 55% to the audio-first condition). The most common reason for nonparticipation was declining interest to participate, followed by difficulties attending the session due to travel or location issues and not meeting the inclusion criteria. Most participants did not meet the inclusion criteria because they scored below the cutoffs for severity of RNT. A total of 3 participants who consented did not attend the session, 2 (67%) due to difficulties with travel and the other (33%) due to enrollment in another study that precluded their participation. All 20 participants who attended the session completed both the VR and audio exercises, assessments, and the interview, indicating a 100% (20/20) retention rate.

Table 1 summarizes the demographic and clinical characteristics of the sample. The sample were mostly in their early 20s; relatively evenly split across male, female, and transgender individuals; and born in Australia, with none identifying as Aboriginal or Torres Strait Islander. Approximately half (8/19, 42%) of the sample were receiving current mental health services, and the other half (11/19, 58%) were not. Clinical scores indicated that the sample was experiencing moderate to severe depression and anxiety, with high levels of RNT. There were no significant differences between groups on any baseline demographic or clinical variable (*P*>.05).

**Table 1:** Sample characteristics (N=20).

			VR^a^-first group^b^ (n=9)		Audio-first group^c^ (n=11)		Total sample
Age (y), mean (SD)			21.33 (2.96)		20.64 (2.66)		20.95 (2.74)
**Gender, n (%)**							
	Male	3 (33)		2 (20)^d^		5 (26)^e^	
	Female	2 (22)		6 (60)^d^		8 (42)^e^	
	Transgender	4 (44)		1 (10)^d^		5 (26)^e^	
	Other	0 (0)		1 (10)^d^		1 (5)^e^	
**Place of birth, n (%)**							
	Australia	7 (78)		6 (55)		13 (65)	
	Africa	0 (0)		1 (9)		1 (5)	
	Asia	2 (22)		2 (18)		4 (20)	
	Europe	0 (0)		1 (9)		1 (5)	
	North America	0 (0)		1 (9)		1 (5)	
**Aboriginal or Torres Strait Islander, n (%)**							
	Yes	0 (0)		0 (0)		0 (0)	
	No	9 (100)		11 (100)		20 (100)	
**Mental health service use, n (%)**							
	Not current service user	5 (56)		6 (60)^d^		11 (58)^e^	
	Current service user	4 (44)		4 (40)^d^		8 (42)^e^	
**Clinical characteristics, mean (SD)**							
	PHQ-8^f^ score	18.33 (2.92)		16.91 (3.33)		17.55 (3.15)	
	GAD-7^g^ score	14.67 (2.65)		14.91 (4.25)		14.80 (3.53)	
	Brief RRS^h^ score	16.33 (2.35)		16.36 (2.46)		16.35 (2.35)	
	RTQ-10^i^ score	45.00 (4.18)		44.00 (4.90)		44.45 (4.50)	
	Brief PSWQ^j^ score	21.89 (2.89)		22.09 (3.05)		22.00 (2.90)	

^a^VR: virtual reality.

^b^Refers to the group of participants who completed the VR exercise followed by the audio exercise.

^c^Refers to the group of participants who completed the audio exercise followed by the VR exercise.

^d^n=10.

^e^n=19.

^f^PHQ-8: 8-item Patient Health Questionnaire.

^g^GAD-7: 7-item Generalized Anxiety Disorder scale.

^h^RRS: Ruminative Response Scale.

^i^RTQ-10: Repetitive Thinking Questionnaire–10.

^j^PSWQ: Penn State Worry Questionnaire.

A summary of the use and familiarity of the sample with VR technology and use of digital technologies for mental health support is provided in Multimedia Appendix 1. These data indicate that the sample had previously used a range of digital technologies to support their mental health; however, none had used VR for therapy previously. Two-thirds of the sample had used VR before, and most were very comfortable, but average use was very infrequent.

### Pretest-Posttest Changes

A 2-tailed repeated measures *t* test was used to explore changes in state-based variables before and after the VR and audio exercises. As this study was not powered to detect effects, it should be noted that patterns of effect sizes across outcomes provide the most meaningful interpretation of changes in clinical measures. As shown in Table 2, for the VR condition, there were large and statistically significant improvements in thought discomfort, cognitive defusion, and state anger. There were moderate but nonsignificant improvements in thought negativity; thought accuracy; thought importance; state rumination; and state tension, depression, fatigue, confusion and vigor, and subjective distress. For the audio condition, there were large and significant improvements in thought negativity, thought discomfort, cognitive defusion, state rumination, state tension, depression, anger and confusion, and subjective distress. There were moderate but nonsignificant improvements in thought accuracy and state fatigue and very small improvements in state vigor. Levels of mindfulness were slightly higher on average in the audio condition than in the VR condition, and this difference was more pronounced for the decentering subscale relative to the curiosity subscale.

**Table 2:** State-based measures before (T1) and after (T2) the virtual reality (VR; n=9) and audio (n=11) exercises with associated statistics.^a^

		Before (T1), mean (SD)	After (T2), mean (SD)	Repeated measures statistics			Cohen *d* (95% CI)
				*t* test (*df*)	*P* value		
**NTA^b^: thought negativity**							
	VR	7.78 (1.39)	5.78 (2.22)	2.00 (8)	.08	0.67 (−0.08 to 1.38)	
	Audio	9.18 (0.87)	6.64 (2.01)	5.37 (10)	<.001	1.62 (0.69 to 2.52)	
**NTA: thought discomfort**							
	VR	8.56 (1.33)	6.11 (2.67)	2.72 (8)	.03	0.91 (0.10 to 1.67)	
	Audio	9.00 (1.18)	5.36 (1.75)	6.90 (10)	<.001	2.08 (0.99 to 3.14)	
**NTA: thought accuracy**							
	VR	7.33 (2.45)	5.78 (3.83)	1.90 (8)	.09	0.63 (−0.10 to 1.34)	
	Audio	7.45 (2.30)	6.18 (2.09)	1.81 (10)	.10	0.55 (−0.10 to 1.17)	
**NTA: thought importance**							
	VR	8.56 (1.88)	7.22 (2.28)	1.60 (8)	.15	0.53 (−0.18 to 1.22)	
	Audio	8.45 (1.81)	7.09 (2.88)	2.01 (10)	.07	0.61 (−0.05 to 1.24)	
**SCFQ^c^: defusion**							
	VR	44.00 (4.72)	31.11 (12.41)	3.02 (8)	.02	1.01 (−0.23 to 1.08)	
	Audio	43.27 (3.82)	26.09 (6.83)	10.31 (10)	<.001	3.11 (1.63 to 4.56)	
**BSRI^d^: rumination**							
	VR	479.67 (201.35)	366.44 (231.47)	1.19 (8)	.27	0.40 (−0.30 to 1.07)	
	Audio	605.00 (106.57)	410.55 (133.94)	6.14 (10)	<.001	1.85 (0.84 to 2.83)	
**POMS^e^: tension**							
	VR	15.44 (2.30)	13.33 (3.84)	1.20 (8)	.27	0.40 (−0.29 to 1.07)	
	Audio	15.18 (3.25)	10.09 (3.65)	7.08 (10)	<.001	2.13 (1.03 to 3.21)	
**POMS: depression**							
	VR	15.33 (3.08)	12.33 (4.33)	2.11 (8)	.07	0.70 (−0.05 to 1.42)	
	Audio	16.18 (3.28)	11.72 (2.83)	5.64 (10)	<.001	1.70 (0.74 to 2.63)	
**POMS: anger**							
	VR	10.78 (1.86)	6.67 (1.66)	4.14 (8)	.003	1.38 (0.43 to 2.29)	
	Audio	9.64 (4.11)	6.00 (2.28)	4.98 (10)	.001	1.50 (0.61 to 2.36)	
**POMS: fatigue**							
	VR	13.22 (5.07)	12.33 (5.22)	1.40 (8)	.20	0.47 (−0.24 to 1.15)	
	Audio	17.09 (2.21)	15.82 (3.57)	1.85 (10)	.09	0.56 (−0.09 to 1.18)	
**POMS: confusion**							
	VR	13.44 (2.70)	11.33 (3.91)	1.51 (8)	.17	0.50 (−0.21 to 1.19)	
	Audio	12.82 (3.71)	9.82 (2.14)	3.93 (10)	.003	1.19 (0.39 to 1.95)	
**POMS: vigor**							
	VR	7.78 (3.49)	9.22 (3.70)	−1.55 (8)	.16	−0.52 (−1.20 to 0.20)	
	Audio	7.09 (2.26)	7.27 (3.17)	−0.18 (10)	.86	−0.05 (−0.64 to 0.54)	
**SUDS^f^: distress**							
	VR	58.56 (30.00)	43.44 (25.58)	1.38 (8)	.20	0.46 (−0.24 to 1.14)	
	Audio	59.36 (18.90)	35.09 (18.16)	4.96 (10)	.001	1.49 (0.60 to 2.35)	
**TMS^g,h^ state mindfulness: decentering subscale**							
	VR	—^i^	24.00 (6.67)	—	—	—	
	Audio	—	26.27 (5.22)	—	—	—	
**TMS** ^g^ **state mindfulness: curiosity subscale**							
	VR	—	21.44 (7.52)	—	—	—	
	Audio	—	22.91 (3.94)	—	—	—	

^a^Positive effect sizes represent improvement.

^b^NTA: Negative Thought Assessment.

^c^SCFQ: State Cognitive Fusion Questionnaire.

^d^BSRI: Brief State Rumination Inventory.

^e^POMS: Profile of Mood States–Adolescents.

^f^SUDS: Subjective Units of Distress Scale.

^g^Completed after each exercise; therefore, there are no data from the “before” time point for this measure.

^h^TMS: Toronto Mindfulness Scale–State Version.

^i^Not applicable.

### User Experience and Satisfaction

Participant feedback on the user experience of the VR and audio exercises is shown in Table 3. Individual item scores suggest that both the VR and audio exercises were rated positively; however, VR tended to be viewed as more fun and engaging but less comfortable, more confusing, and less of a learning experience compared to the audio condition.

**Table 3:** Participant feedback for the virtual reality (VR) and audio exercises from the user experience questionnaire (N=20).^a^

	VR exercise, mean (SD)	Audio exercise, mean (SD)
“I found the experience enjoyable.”	4.11 (1.05)	4.27 (0.65)
“I found the experience fun.”	4.56 (0.53)	3.64 (0.93)
“I found the experience comfortable.”	3.78 (1.09)	4.45 (0.82)
“I found the experience interesting.”	4.56 (0.53)	4.73 (0.47)
“I found the experience engaging.”	4.44 (0.73)	4.27 (0.65)
“I found the experience easy to do.”	4.33 (0.87)	4.27 (1.01)
“I learnt something from the experience.”	3.56 (1.42)	4.55 (0.52)
“I would like this sort of experience to be a part of therapy.”	4.11 (1.05)	4.45 (0.69)
“I would be likely to do this experience in my own time.”	4.22 (0.97)	4.36 (0.67)
“I would be motivated to do this experience again.”	4.33 (0.71)	4.64 (0.67)
“I would recommend the experience to other people with depression and/or anxiety.”	4.33 (0.87)	4.64 (0.67)
“I found the experience confusing.”	2.44 (1.59)	1.45 (0.52)
“I found the experience boring.”	1.11 (0.33)	1.55 (0.69)
“I found the experience upsetting or distressing.”	2.22 (1.48)	2.00 (0.89)

^a^Scale range from 1 (*strongly disagree*) to 5 (*strongly agree*); ratings were pooled across the entire sample regardless of the order of the exercises.

### Level of Presence in VR

Table 4 shows the subscale scores for the ITC-SOPI. The pattern of scores supported a moderate to high level of spatial presence, indicating that participants felt a reasonable degree of “being there” in the virtual environment. The highest score was for engagement, indicating that participants felt moderately involved and interested in the VR experience. Ecological validity and naturalness was relatively low, suggesting that, while the VR environment was somewhat convincing and behaved in a way that users expected, there could be improvements in making the environment and interactions feel more realistic. The low average score on negative effects suggests that participants generally did not experience significant discomfort, disorientation, or other adverse effects while using the VR application, supporting that it was well tolerated.

**Table 4:** Subscale scores on the ITC–Sense of Presence Inventory.^a^

Subscale	Scores, mean (SD)
Spatial presence	3.32 (0.79)
Engagement	3.82 (0.55)
Ecological validity and naturalness	3.07 (0.97)
Negative effects	1.81 (0.72)

^a^Scale range from 1 (*strongly disagree*) to 5 (*strongly agree*).

### Qualitative Feedback

All 20 participants provided feedback in a qualitative interview after they had completed both the VR and audio exercises. The themes arising from these interviews and representative quotes are described in the following sections.

#### VR Is “New, Different, and Engaging”

I prefer the VR one mainly because...it’s something I don’t think I’ve ever really tried before and it’s something new and different and engaging.

Participant 51; male; aged 19 y

All young people interviewed (20/20, 100%) reported preferring VR over the audio exercise. Reasons for this preference centered on VR being perceived as a more engaging, novel, and interesting way of learning psychological techniques. Young people were interested in the novelty of VR as “something that I haven’t really done before” (participant 91; male; aged 21 y), “just a whole different perspective” (participant 91; male; aged 21 y), and a “very interesting and new type of therapy” (participant 42; female; aged 25 y). This uniqueness was perceived as “new and fresh” (participant 61; male; aged 21 y), “fun” (participant 66; nonbinary; aged 20 y), “cool” (participant 72; female; aged 19 y), and “progressive” (participant 44; female; aged 21 y) relative to the audio, which was seen as “nothing new” (participant 61; male; aged 21 y). It was described as “very important to attract young people’s interest” (participant 42; female; aged 25 y) as a way of “getting young people to engage with their mental health” (participant 91; male; aged 21 y). In addition to the novelty of VR, the “fun” (participant 46; other; aged 16 y) and “enjoyable” (participant 66; nonbinary; aged 20 y) aspects of the experience were also seen as an important way of engaging young people:

...being able to do something fun as well as therapy, like it makes it a lot better in my opinion.

Participant 85; female; aged 16 y

#### VR Facilitated Cognitive Defusion

...being able to have this distressing thought and then turn it into something that seemed really not important, and being able to interact with it in a way where I can really visualise it...I think it just sort of helps with that, compartmentalizing of thoughts.

Participant 64; female; aged 24 y

Consistent with the aims of cognitive defusion, some young people described that the act of physically interacting with their thoughts made it easier to separate from them:

It’s kind of harder to see it as a fact when it when you can like so easily manipulate it.

Participant 44; female; aged 21 y

Key to this was the interactive and visual element, where they could “see and actually like interact, rather than just sit there and try to imagine” (participant 86; female; aged 17 y).

The process of physically acting out the technique was described as “easier” (participant 71; female; aged 24 y) compared to “mindfulness stuff where you’re just listening, you’re just like, I feel like it takes a lot of like activation energy to get yourself to do it. Whereas with the VR, like throwing for example like you’re just throwing and the mindfulness comes like secondarily” (participant 61; male; aged 21 y). However, many young people saw value in the audio exercise as well, with both offering different outcomes:

I think the audio exercise was more relaxing and then the VR exercise was more engaging and, yeah so two different things, and they had two different outcomes.

Participant 91; male; aged 21 y

Young people also described that the immersive environment helped them focus more actively on the exercise with less distraction:

I just had a fun time and wasn’t distracted at all. I was paying a lot of attention.

Participant 91; male; aged 21 y

Some found that this meant that they “could learn a lot more” (participant 85; female; aged 16 y). Some felt that this greater focus meant that VR was “probably a little bit more effective” (participant 44; female; aged 21 y) because it enabled them to be “totally involved” (participant 44; female; aged 21 y), whereas “during the audio, I still had like random thoughts coming to mind and you know, partly focusing on that, partly focusing on the audio, but in the VR I was like totally in the VR” (participant 44; female; aged 21 y).

#### Gamification May Create Disconnection From the Technique

VR could potentially be distracting, that you’re in a game and you’re more like, oh, this is a game rather than doing therapy.

Participant 46; other; aged 16 y

Some young people questioned whether the gamified element undermined the therapeutic intent of the VR experience. The opportunity for distraction from thoughts had appeal but risked not addressing the core issue:

I do like to be distracted by my thoughts often and kind of like, yeah, even though it doesn’t fix the problem.

Participant 72; female; aged 19 y

The generalizability of the skill beyond the immediate experience into everyday situations was also questioned by 10% (2/20) of the young people:

...how much crossover is there between when you do that with the VR headset...and being able to take that away and then still be get into that visualising sense without it.

Participant 61; male; aged 21 y

...whenever I do get a negative thought, I just can’t throw it or pop it.

Participant 86; female; aged 17 y

#### Implementation Considerations

This is an experience that I’d want to do again in the future and in a therapeutic setting.

Participant 91; male; aged 21 y

Young people supported the idea of the VR application being used within therapy, describing it as “something that would be very interesting to a lot of people, especially young people” (participant 91; male; aged 21 y). The appeal of using the VR application within treatment centered on the opportunity to “get [young people] through the door” (participant 91; male; aged 21 y) and “probably make them inclined to stay because of how interesting it is” (participant 91; male; aged 21 y). Some also mentioned the potential for the VR application to help strengthen the therapeutic relationship by creating “common ground” (participant 61; male; aged 21 y) as the young person was able to talk through their interactions within the VR. However, the need for appropriate supports for both young people and clinicians was mentioned, including training and guidance on how to introduce and support young people in using VR more generally, such as a “PDF that they have on hand” (participant 91; male; aged 21 y). Young people also warned that it would “take some time to get familiar with it” (participant 42; female; aged 25 y).

The biggest perceived barrier to using the VR application was the cost of VR headsets and production of the software:

The only downside that I can see is that it costs money to do. Yeah, since VR isn’t very cheap.

Participant 91; male; aged 21 y

Challenges accessing the equipment were mentioned in terms of both home use and clinical settings, with a need for “centres and clinicians being able to get access to VR headsets and set them up and keep up with the IT and the maintenance” (participant 61; male; aged 21 y).

A number of young people reported that the VR application may be particularly well suited to specific clinical populations, particularly those with attention difficulties or for whom abstract mental thought was challenging. They reiterated the appeal of the VR application for young people with depression and anxiety who might benefit from separation from thoughts:

I think a lot of people like struggling with depression anxiety could sometimes be like yeah, angry at themselves or angry at other people, and it just was very like satisfying being able to like, let that go.

Participant 72; female; aged 19 y

The VR application was also seen as potentially beneficial for young people with attention difficulties as a way to facilitate focus without distraction:

I think that VR could be a lot better for young people who suffer with ADHD and other types of attention deficits, especially since sitting there for 10-15 minutes might be hard for people who are a bit restless.

Participant 91; male; aged 21 y

#### Future Changes to the VR Application

When asked directly what changes they would make to the VR application, most young people reported minor suggestions. This included expanding the range of exercises, including “visualisation activities...could really be awesome, like the classic, like, you have a negative thought and just stick it on a cloud. And see it go away. You can literally make that” (participant 61; male; aged 21 y). Several young people also suggested combining the audio and VR exercises to gain the added benefit of both:

...the headset itself like provides that sort of like audio guidance would be nice.

Participant 64; female; aged 24 y

Improvements to the user interface were mentioned as a priority, including clearer instructions for controls and interactions. Many young people suggested expanded options for personalization. As there were only 3 options of environments to choose from, some young people suggested that “there might be like some people that like don’t really resonate with any three of them” (participant 72; female; aged 19 y) and that the environments could be “a little more realistic to my situation” (participant 44; female; aged 21 y), with suggestions to add “more options” (participant 71; female; aged 24 y) such as “a university classroom rather than like a school” (participant 44; female; aged 21 y). One young person suggested that nonrealistic environments that draw on traditional visualizations, such as representing thoughts as clouds, may also be appealing.

Some commented that the characters and environments could appear more realistic, with some avatars described as “a little bit creepy looking” (participant 61; male; aged 21 y), which could break immersion:

I just found the people really weird and just sort of took me out of it because they were doing very stiff or weird sort of motions.

Participant 51; male; aged 19 y

However, most young people found the graphic style acceptable.

#### Safety and Adverse Events

One young person experienced some distress during the VR exercise and requested to stop. This participant found the experience of manifesting a distressing thought in VR confronting; however, they were able to continue and complete the postassessment and interview at their request following a break and some reassurance. They were contacted the following day and reported no persisting discomfort or distress. No other participants reported adverse events during the study. In the qualitative interview, negative experiences of the VR application were mentioned by 10% (2/20) of the young people. One described feeling “a bit panicky and anxious in the VR experience, but it was OK to like kind of push through” (participant 67; nonbinary; aged 22 y). Another young person described that “thinking about the thought was quite distressing, but it was also helpful with the interactive bit” (participant 94; male; aged 24 y). No participants reported experiencing any cybersickness.

## Discussion

### Principal Findings

This study explored the potential of using VR to support third-wave CBT [18] for young people with depression and anxiety. To achieve this, a prototype VR application was developed, which involved a brief, interactive cognitive defusion exercise within personalized virtual environments. Using a mixed methods experimental approach, this study sought to investigate the feasibility, acceptability, usability, and safety of the VR application; explore whether it could improve negative thinking and mood states; and understand young people’s experiences of this new VR treatment approach. Participants in this study completed both the VR and audio defusion exercises within a single session in a randomized order to enable an understanding how these experiences compared. Key results indicated that the VR application was feasible, safe, and acceptable to use. However, the pattern of effects across the 2 conditions suggested that the audio exercise may have led to greater immediate improvements in certain mood states despite qualitative themes indicating that VR was preferred by participants.

While the small sample precludes generalizable interpretations, the results of this study suggest that a single session of VR cognitive defusion can lead to reduced discomfort with negative thoughts and state anger and increases in cognitive defusion. This provides proof-of-concept evidence that the application can engage the target mechanism of cognitive defusion (pretest-posttest effect size=1.01). Acceptability ratings indicated that the intervention was perceived to be enjoyable and the environment was immersive and engaging, with the experience well tolerated with minimal adverse effects. Qualitative themes supported these quantitative results, with young people reporting that the physical interaction and visualization of VR provided a more concrete and interactive learning experience that helped them separate from their negative thoughts and that it was more fun and interesting compared to the audio exercise. Young people reported that these benefits may be particularly important for those who struggle with understanding abstract therapeutic concepts, such as those with autism spectrum disorders, or those who have attention and concentration difficulties such as attention-deficit/hyperactivity disorder. These perspectives align with literature emphasizing the need to tailor third-wave therapy approaches to the cognitive developmental stage of adolescents [28]. This is also consistent with the perspectives of clinicians and individuals with lived experience of developmental disorders, who advocate for novel therapeutic strategies that better accommodate sensory needs and learning styles, including VR [34]. This includes adapting exercises to be interactive, experiential, creative, interesting, and behaviorally focused. Both qualitative themes and acceptability survey responses suggest that VR can be used to support these adaptations and that this is valued by young people.

Despite the preference among young people for the VR application and its positive effects on some outcomes, effect sizes were larger in the audio condition. While the small sample size prevented between-group comparisons and, overall, the interpretation of results must be approached with caution, this finding suggests that the defusion exercise delivered via audio may have led to greater improvements in mental states than those achieved with VR. Previous research on VR-based cognitive defusion is limited, with only 1 study to the authors’ knowledge by Prudenzi et al [30] finding that a VR defusion task reduced thought believability and discomfort compared to a nondefusion VR control. Our study also showed improvements in these domains for the VR defusion task; however, the audio showed more favorable effects. One explanation for this difference is the choice of comparator. In this study, the control condition was a non-VR, audio-based defusion task that produced stronger effects, suggesting that audio guidance may be more effective in facilitating cognitive defusion. Other relevant studies have examined VR-guided meditation, finding that users tend to prefer VR over nonimmersive formats (eg, audio, video, or text) due to its novelty, immersion, and engagement [54-56]. However, unlike our findings, VR-guided meditation has generally been associated with greater improvements in positive mood states than non-VR meditation, with less consistent effects on negative mood states. One possible explanation is that VR’s sensory richness may support mood regulation in meditation by enhancing absorption and presence in the environment, whereas it may increase cognitive load and interfere with techniques such as cognitive defusion that require more cognitive effort to achieve abstract mentalization. Indeed, previous research [57] has found that the novelty of VR can hinder learning by increasing cognitive load as users adjust to the unfamiliar interface and immersive experience, potentially diverting attention from the intended task. Future research should explore ways to optimize VR-based defusion, such as simplifying visuals, adjusting interactivity, and selecting or tailoring virtual environments and scenes.

Another possible explanation for these findings is that the way in which the exercises were delivered influenced how participants engaged with their thoughts, particularly in terms of cognitive focus and mental manipulation. Cognitive defusion and related third-wave CBT techniques typically encourage individuals to sit with uncomfortable thoughts and view them as separate to the self [19-21]. This is a highly reflective process that requires individuals to focus and mentally manipulate their thoughts using their imagination. The audio exercise may have been more effective at achieving this due to the lack of external distractions, forcing participants to focus their attention on the thought. In contrast, the VR exercise involved creating a visual object to externalize the thought within a virtual environment and interacting with it in gamified ways. By externalizing the thought so directly, individuals may have lost the connection between the internal representation of their thought and, instead, related to the object as an external entity, thus losing the level of focus and mental manipulation required for successful cognitive defusion. Furthermore, the gamification element may have counterproductively enabled participants to experientially avoid their negative thoughts and further sever the connection between the object and their internal representation. This theory is supported by qualitative interview themes, in which some participants described the VR exercise as potentially being a fun distraction, which may have undermined therapeutic learning, as well as by the description of the audio requiring “more energy.” This explanation suggests that, for the VR cognitive defusion exercise to be effective, individuals must maintain a connection between the symbolic thought in the virtual environment and its internal representation for them to effectively internalize the experience.

Another explanation for the findings is that the level of guidance and explanation within the VR exercise was insufficient to support young people in applying the skill of cognitive defusion in the moment. Ratings on the acceptability measure suggest that the audio condition provided a better learning experience compared to the VR condition. Given that VR is a highly engaging visual medium, participants may have become either too distracted or potentially overwhelmed by the exercise in a way that reduced their focus on the core exercise, thus undermining their learning. This aligns with aforementioned research indicating that the novelty of VR can hinder learning, suggesting the need for a prelearning phase to help users acclimate to the environment before therapy or a structured approach to gradually build toward more complex tasks [57]. Indeed, a small number of young people did report becoming distressed by the experience in VR. While this did not appear to have a lasting effect, it may have undermined their ability to engage with the exercise. Other factors that may explain these effects could be the dosage as a single session may be insufficient to induce effects for VR, as well as a potential “delayed” response in which effects might be observed for a longer period than in the audio exercise. Indeed, participants described VR as being more fun and exciting compared to the audio, which was more relaxing. It might be that the audio achieved its therapeutic benefits by lowering arousal, leading to more immediate improvements in mood states. In contrast, given the level of immersion and engagement in the VR experience, participants may have been left feeling more activated. This is supported by the effect of the VR application on increasing vigor, whereas the audio did not show such effects on this outcome. In contrast, the audio had a much stronger effect on reducing tension. Thus, the immediate aftereffects of VR might not be the ideal time frame to uncover beneficial outcomes.

Despite these findings, young people in this study clearly endorsed the VR application as a valuable clinical tool, with all young people reporting that they preferred it over the audio experience. A central theme to this preference was the novelty of VR and the opportunity to engage young people in treatment using a new and interesting approach. This is consistent with a previous study examining the addition of VR within a mindfulness intervention for generalized anxiety in adults, which found that those who received the addition of VR were more likely to complete the intervention compared to those who did not [31]. Given that engagement with treatment is a key challenge in youth mental health care [11], this opportunity is significant. However, in considering the contrasting findings indicating that the audio condition yielded greater effects despite VR being preferred by participants, it is important to recognize the potential novelty effect of VR. The initial appeal of VR may contribute to a “digital placebo” effect [58] where therapeutic effects and engagement benefits stem from the technology itself rather than the therapeutic content. Moreover, while novelty may drive short-term engagement, its impact may diminish over time, potentially reducing sustained effectiveness. Such novelty may have a greater impact on those with less VR experience and could be examined by comparing engagement across varying levels of previous VR exposure. Although we did not examine differences in engagement between participants with previous VR experience and those new to VR given sample size limitations, future research should investigate how previous exposure influences engagement and therapeutic outcomes. Studies incorporating nonactive VR comparators (eg, VR games) will also help disentangle the effects of novelty from the core therapeutic mechanisms, informing strategies to sustain engagement beyond the initial novelty phase.

Young people reported being particularly interested in using the VR application within a therapy session delivered by a clinician; however, they also reported a need for clear guidance and clinical protocols. This is consistent with qualitative studies [59,60] of clinician attitudes toward VR that emphasize the need to address implementation barriers, as well as with broader literature on the importance of working with end users to ensure that technologies are fit for purpose and appropriately designed for the implementation context [2,61]. In addition to opinions about the use of the VR application in clinical settings, young people also called for improvements to the user experience. This included an expansion to the range of activities and environments, improvements to the graphics and user interface, and more options for personalization. This study’s findings also suggest that there should be improvements to the guidance offered within the experience to better support therapeutic learning. For example, several young people reported enjoying both VR and the audio exercises and suggested that a combination of both would be optimal.

In considering future iterations of the VR application, there may also be benefit in refining the therapeutic target. Specifically, the application may be better positioned as a treatment targeting decentering as a core underlying mechanism rather than an adaptation of cognitive defusion. Decentering is a broader concept that includes cognitive defusion and refers to the process of mentally separating oneself from one’s thoughts and emotions, becoming aware of underlying thinking processes, and reducing emotional reactivity to mental events [22,23]. Decentering has been identified as a core component of psychological interventions for anxiety and depression by reducing the negative emotional impact of stressors, including inner events such as thoughts, feelings, and memories. Indeed, interventions that improve decentering-related processes have been shown to decrease depression and anxiety severity [22]. Decentering techniques typically rely on experiential exercises drawn from third-wave approaches, which includes cognitive defusion as well as mindfulness and ACT. This collection of techniques share an experiential learning focus that may be well suited to VR due to the ability to physically separate and visually manipulate mental processes in an immersive way. Broadening the VR treatment package to include a wider range of techniques to achieve decentering may provide a clearer theoretical model for the intervention while offering additional learning opportunities that can be tailored to the specific needs and preferences of young people.

### Limitations

Future research would benefit from addressing the limitations of this study design. Specifically, a larger trial with a bigger sample size and a more established intervention protocol over a longer period is needed to thoroughly evaluate effectiveness. This may include increasing the number of sessions and incorporating follow-up assessments to examine long-term effects. In addition, the protocol could involve delivering the VR intervention in a blended format, aligning with the perspectives of young people in this study, and exploring how the intervention might be implemented in routine clinical practice. A hybrid implementation-effectiveness trial [62] that simultaneously tests both implementation and effectiveness outcomes would be ideal for establishing evidence and fast-tracking implementation in real-world settings. Notably, the conversion rate suggests that only a proportion of young people who are eligible may be interested in the VR application, suggesting that a clearer understanding of who the approach is best suited for is needed. This may involve expanding the age range of the VR offering from 16 to 25 years to 12 to 25 years, in line many with youth mental health services in Australia, to understand whether this type of intervention would be more appealing to a younger demographic. Another factor to consider in future trials is the selection of a control group. The active comparison of an audio cognitive defusion exercise with a repeated measures design in this study was a strength, providing insights into how VR compares to traditional methods. However, carryover effects from the first condition to the second prevented direct comparisons, and the chosen control group did not isolate specific VR-related effects. Furthermore, as previously mentioned, it is important that future research address the potential “digital placebo” effect [58] involved in VR by including control groups with a therapeutically inactive VR experience to better isolate the intervention’s effects. Other comparison groups of interest would include a passive control group or an alternative non–VR-based intervention to further clarify the distinct effects of VR-based therapy. Finally, limitations should be noted regarding the measures collected in this study. We created a specific measure of acceptability based on the need to capture more nuanced aspects of user experience relevant to this VR intervention. However, future research should incorporate validated measures of acceptability of digital mental health platforms alongside study-specific measures where possible. Furthermore, it is possible that participants experienced fatigue effects from the number of questionnaires completed in a single session. While no participant reported such fatigue, future research should examine this by analyzing consistency in the responses.

### Conclusions

In conclusion, the findings of this study indicate that the VR cognitive defusion application is feasible, safe, and acceptable to young people and shows potential for supporting mental health treatment by increasing engagement and offering a novel, enjoyable approach to skill development within third-wave CBT approaches. The greater immediate improvements observed in the audio condition compared to the VR condition suggest that the application needs to be refined to optimize the learning experience. Future research should focus on improving the overall experience based on young people’s feedback and conducting a larger trial with an established intervention protocol within a real-world implementation setting.

## Appendix

Multimedia Appendix 1 Scripts used in the study and additional demographic information.

## Abbreviations

ACT: acceptance and commitment therapy
Brief PSWQ: Brief Penn State Worry Questionnaire
Brief RRS: Brief Ruminative Response Scale
BSRI: Brief State Rumination Inventory
CBT: cognitive behavioral therapy
GAD-7: 7-item Generalized Anxiety Disorder scale
ITC-SOPI: ITC–Sense of Presence Inventory
NTA: Negative Thought Assessment
PHQ-8: 8-item Patient Health Questionnaire
POMS-A: Profile of Mood States–Adolescents
RNT: repetitive negative thinking
RTQ-10: Repetitive Thinking Questionnaire–10
SCDQ: State Cognitive Fusion Questionnaire
SUDS: Subjective Units of Distress Scale
TMS: Toronto Mindfulness Scale
VR-CBT: virtual reality–based cognitive behavioral treatment
VR: virtual reality

## Data Availability

The datasets generated or analyzed during this study are available from the corresponding author on reasonable request.
