# Supplementary material for: A Virtual Reality–Based Cognitive Defusion Application for Youth Depression and Anxiety: Mixed Methods Experimental Study
Source: JMIR Ment Health. 2025 Aug 7;12:e70160. doi: 10.2196/70160 (PMC12371279; doi:10.2196/70160)
Supplement: Multimedia Appendix 1 [file mental_v12i1e70160_app1.docx]

**Supplementary 1 – scripts used within study**

***Defusion preamble prior to commencing first exercise***

*The way we think affects our mood. We can often think in negative ways. We can have negative views of ourselves, such as“I’m no good”, the world (e.g. “Life has no meaning”) and the future (e.g. “I will always feel this way”).*

*However, imagine if we saw thoughts in a different way. Another perspective is that the mind is a great storyteller. It tells stories about what we notice, who we are and aren’t, of who we’d wish to be and who we must not be. Sometimes these stories are true in the sense that someone else would agree, or the thought might correspond to objects or events we can see. We often call these thoughts “facts.”*

*But most thoughts are hard to prove. They are evaluations, judgements, attitudes, morals, theories and dreams. What if we stop asking if the thought is “true”” and start asking if it’s helpful? To do this we need to learn how to take a step back from the thoughts.*

*There are techniques that can help us step back from our thoughts and see them for what they are, words and images that form a continuous story told by our mind. We call the goal of these techniques diffusion.*

*You are going to have a go at two different diffusion exercises, one in VR and another in audio. These exercises might help you to disconnect from the negative thought you chose earlier.*

*The VR and audio experiences are going to be quite different, but they will both involve trying to look at the negative thought in a different way so that’s it’s less upsetting.*

***VR defusion exercise script***

*In a moment, you’ll put on the VR headset. You’ll see a space and you can move your head around to see what’s around you. You will use these hand controllers to navigate your way through the different options (show buttons). I’ll guide you though it all, don’t worry, and remember that you can take the headset off at any time you need to. Some people feel a little funny when they first go into VR, and that’s OK. It might take some time to get used to it.*

[Puts on headset and gives instructions for hand controllers]

*Right now, you’ll see a menu with different environments. There’s a classroom, a bar, and a train carriage.*

*This VR experience helps you to practice a technique to make negative thoughts, like the one you chose earlier, less upsetting. Because these thoughts tend to be triggered in certain environments, you can choose which environment is most relevant to your negative thought. When you think about the negative thought you chose earlier that really bothers you, which one of these environments do you think that thought is most likely to come up?*

[Guide the participant to select an environment most relevant to them. If none of them are relevant, get them to choose one that they think is most relevant]

*Use the hand controller to click into that environment Take a look around, you should now be in the environment. Take a moment to think about the negative thought that you struggle with in this environment. Thinking about the thought might bring about some feelings, such as sadness or anger. That is normal because thoughts are what leads us to feel things. We might feel angry if we think someone has done something to hurt us, or sad if we think we might have failed at something.*

*Notice how your thought comes to you in this environment. Is something going on around you that is bringing this thought to mind? What emotions come up for you here? Just sit with that for a moment.*

*The idea behind this exercise is to take that thought out of your mind and into the environment around you. Then, we are going to play with that thought a bit by changing the way it looks and feels. The thought might be uncomfortable to sit with, and that’s OK. Try not to struggle with the thought, just see it as something outside of you for a little while.*

*In front of you, there is an object floating. Take a moment to think of this object as the negative thought you have. Notice where it is in the environment and that it’s no longer in your head, but outside.*

*Point the controller at the object and press the button at the back of the controller. This opens a menu.*

*The first step is to write some words in the object to represent that thought. If you could summarise your negative thought in a few words, what would it be? Use the keyboard to type the words into the thought. Now the thought is in front of you, not inside your head.*

*Now click on the thought in front of you again to open the menu. Now you can change the shape of the thought. When you think about the thought, what shape represents it best? What made you choose that shape?*

*Select the shape and then observe it for a moment. Does is have sharp edges or is it smooth? Where is the shape in the environment? Is it blocking your view of things behind it? That’s a bit like thoughts in the real world. They can get in the way of us seeing things for how they really are when we get really attached to them.*

*Now go back to the menu and change the colour of the thought. You can change it to whatever colour you like. What colour did you choose? What made you choose that colour?*

*Notice the way the thought looks to you in the environment you are in. You can change the colour if you like, noticing how it changes the way the thought looks and feels.*

*Use your hands to take hold of the object and move it around. Then pull to expand the size of the shape. You can make it really big or really small.*

*Notice where the shape is in the environment. Can you block your view by making it very large or see more clearly by making it small?*

*You might want to put it behind you or to the side, or rest it on the ground. We can do this with our thoughts, choosing to move them out of our main focus so we can concentrate on other things.*

*Go to the ‘float me’ option in the menu. This will make the object float around. Whilst it’s floating, you can choose to look away from the thought if you want to. Tune in to other things that are around you. Are there people or things? Notice these things and let the thought continue to float around without changing it at all. We can choose to pay attention to what we want to – the thought might still be there, but you don’t have to pay attention to it if you don’t want to.*

*Now go back to the menu and press ‘pop me’. This will make the thought float around you in different places. It might be behind you, in front, or either side. Use the hand controller to pop the thoughts as they come up. You will get points for popping them more quickly!*

*Each time you pop the shape, it reappears in a different place. This is a bit like what thoughts do, they come back again and again, and that’s OK. The thought is not your enemy, it’s just something that exists around you. You can choose how you interact with it.*

*Once the game ends, tune into your emotions for a moment. What do you feel in this moment? Have there been any changes since you started the exercise? Does the thought feel as upsetting as it did before?*

*Bring your awareness to your surroundings. Let the thought drift away. When you are ready, take the headset off.*

***Audio defusion exercise scrip***

*Sit back in your chair and get comfortable. Close your eyes and take a few deep breaths.*

*Try to tune out whatever is going on around you and focus on any thoughts and feelings that come to mind.*

*Take a moment to think about the negative thought that upsets you.*

*Thinking about the thought might bring about some feelings, such as sadness or anger.*

*That is normal because thoughts are what leads us to feel things. We might feel angry if we think someone has done something to hurt us, or sad if we think we might have failed at something.*

*The idea behind this exercise is to let thoughts and feelings come and go, without getting so caught up in them. Not getting tangled up in them or trying to control them, but just letting them be.*

*To do this, it can help to think about our thoughts in a different way.*

*Is there a particular place where your negative thought comes to you the most? Maybe it’s in class, a restaurant or a train. Wherever your negative thought happens most, bring this place to mind.*

*Now imagine you are in this place. Notice your surroundings and what is going on around you. Are there people there or things that are familiar to you? Imagine you are an observer in this place, watching things happen without*

*Notice how your thought comes to you here. Is something going on around you that is bringing this thought to mind? What emotions come up for you here?*

*Keep this place in mind as we look a bit closer at this thought.*

*Take a moment to imagine the thought as an object in this environment.*

*Try to visualise the thought as something physical, like a balloon or a box. Notice the shape of the object. Does is have sharp edges or is it smooth? Where is the shape in the environment? Is it blocking your view of things behind it?*

*Now change the colour of the object. You can change it to whatever colour you like. Imagine a rainbow of colours and select one that represents the thought the best. Is it blue, green, yellow or red? Notice the way the thought looks to you in the environment you are in. You can change the colour if you like, noticing how it changes the way the shape looks and feels.*

*Use your hands to take hold of the object and move it around. You might want to put it behind you or to the side, or rest it on the ground.*

*Use your hands to grab a hold of the object. Then pull to expand the size of the shape. You can make it really big or really small.*

*Notice where the shape is in the environment. Can you block your view by making it very large or see more clearly by making it small?*

*Play around with the size and shape of the object, moving it around in your mind.*

*Pick it up and move it around you, changing the colour and shape again if you like.*

*Now place the object in font of you.*

*Imagine you look down and see a pointer stick in front of you. Imagine picking up the pointer with your hand and holding it. Press the end of the pointer into the object to pop it, like a game.*

*Imagine the object floating around you in different places. It might be behind you, in front, or either side. Each time you pop the shape, it reappears in a different place. The thought will come back again and again, and that’s OK.*

*The thought is not your enemy, it’s just something that exists around you. You can choose to look away from the thought if you want to. Tune in to other things that are around you. Are there people or things? Notice these things and let the object continue to float around without popping it.*

*Tune into your emotions for a moment. What do you feel in this moment? Have there been any changes since you started the exercise? Does the thought feel as upsetting as it did before?*

*Bring your awareness to your surroundings. Let the thought drift away. When you are ready, open your eyes.*

**Supplementary 2.** Sample use and familiarity with virtual reality and other technologies

|  | **VR first group (n=9)** | **Audio first group (n=11)** | **Total sample (n=20)** |
| --- | --- | --- | --- |
|  | *n (%)* | *n (%)* | *n (%)* |
| **Technology ownership** *n (%)* |  |  |  |
| *Smartphone* | 9 (100.0%) | 11 (100.0%) | 20 (100.0%) |
| *Laptop* | 8 (88.9%) | 11 (100.0%) | 19 (95.0%) |
| *Desktop* | 7 (77.8%) | 2 (18.2%) | 9 (45.0%) |
| **Hours of technology use per day** *Mean (SD)* |  |  |  |
| *Smartphone* | 6.00 (2.65) | 5.82 (1.72) | 5.90 (2.13) |
| *Internet* | 9.22 (4.35) | 7.91 (2.30) | 8.50 (3.35) |
| *Social media* | 4.00 (3.00) | 3.00 (1.12) | 3.45 (2.17) |
| **Prior use of technology for mental health support**  *n (%)* |  |  |  |
| *Email* | 5 (55.6%) | 6 (54.5%) | 11 (55.0%) |
| *Text* | 3 (33.3%) | 6 (54.5%) | 9 (45.0%) |
| *Smartphone* | 5 (55.6%) | 8 (72.7%) | 13 (65.0%) |
| *Phone* | 6 (66.7%) | 9 (81.8%) | 15 (75.0%) |
| *Videophone (including Facetime, Skype and video conferencing)* | 7 (77.8%) | 9 (81.8%) | 16 (80.0%) |
| *Online information* | 5 (55.6%) | 6 (54.5%) | 11 (55.0%) |
| *Online forum/community/message board* | 4 (44.4%) | 3 (27.3%) | 7 (35.0%) |
| *Online mental health treatment* | 4 (44.4%) | 5 (45.5%) | 9 (45.0%) |
| *Mental health app* | 6 (66.7%) | 7 (63.6%) | 13 (65.0%) |
| **Prior use of virtual reality** *n (%)* |  |  |  |
| *Yes* | 8 (88.9%) | 6 (54.5%) | 14 (70.0%) |
| *No* | 1 (11.1%) | 5 (45.5%) | 6 (30.0%) |
| **Prior use of virtual reality for treatment**^c^ *n (%)* |  |  |  |
| *Yes* | 0 (0%) | 0 (0%) | 0 (0%) |
| *No* | 8 (100%) | 6 (100%) | 14 (100%) |
| **Frequency of use of virtual reality**^c^ *n (%)* |  |  |  |
| *Very rarely* | 3 (38%) | 5 (83%) | 8 (57%) |
| *Monthly* | 3 (38%) | 0 (0%) | 3 (21%) |
| *Weekly* | 1 (13%) | 1 (17%) | 2 (14%) |
| *Once a week or more* | 1 (13%) | 0 (0%) | 1 (7%) |
| **Comfort with virtual reality** *n (%)* |  |  |  |
| *Very uncomfortable* | 0 (0%) | 0 (0%) | 0 (0%) |
| *Uncomfortable* | 0 (0%) | 1 (17%) | 1 (7%) |
| *Comfortable* | 3 (40%) | 3 (50%) | 6 (43%) |
| *Somewhat comfortable* | 1 (13%) | 1 (17%) | 2 (14%) |
| *Very comfortable* | 4 (50%) | 1 (17%) | 5 (36%) |

^c^Prior users only
